# Cutting-edge and topical issues in the treatment of breast cancer with traditional Chinese medicine based on CiteSpace bibliometric analysis

**DOI:** 10.1097/MD.0000000000040784

**Published:** 2024-12-06

**Authors:** Jiapeng Chai, Nan Zhang, Tie Li, Hailin Jiang, Jinying Zhao, Xuefeng Li, Heran Wang, Jiaxun Zhang, Lin Wang, Qi Zhang, Yuxin Jiang, Fuchun Wang

**Affiliations:** a School of Acupuncture and Tuina Massage, Changchun University of Chinese Medicine, Changchun, Jilin, China; b School of Pharmacy, Changchun University of Chinese Medicine, Changchun, Jilin, China.

**Keywords:** bibliometric analysis, CiteSpace, female breast cancer, traditional Chinese medicine, VOSviewer

## Abstract

**Background:**

Breast cancer poses a significant health threat to women, marked by high incidence and mortality rates. Although modern treatment primarily involves surgery and chemotherapy, which may reduce quality of life, limited analysis exists on the effects of traditional Chinese medicine (TCM) on this aspect. In this paper, bibliometric software was used to study the literature related to TCM in the treatment of breast cancer to provide researchers with new insights and directions for development.

**Methods:**

By using CiteSpace and VOSviewer, we conducted an analysis of literature on TCM in breast cancer treatment from the Web of Science Core Collection (2013–2023). The assessment encompassed countries, institutions, journals, authors, keywords, and references, providing a comprehensive overview of developments in the field.

**Results:**

Analyzing 7419 articles, this study revealed an increasing trend in TCM research on breast cancer from 2013 to 2023. China and its institutions, particularly Beijing University of Chinese Medicine, made significant contributions. Liu Zhishun emerged as the most prolific author, while Y. Macpherson received the highest number of citations. The journal *Evidence-Based Complementary and Alternative Medicine* received the highest number of citations. Research primarily centers on TCM approaches for enhancing breast cancer patients’ quality of life and elucidating their underlying mechanisms.

**Conclusion:**

Numerous studies have investigated TCM in treating female breast cancer, revealing treatment trends, analyzing major research topics, currently focusing on acupuncture, breastfeeding, and TCM, which have a greater impact on positively ameliorating breast cancer. Influential authors and publications were also identified. These insights provide valuable guidance for future research, contributing to the foundation for developing effective TCM strategies for breast cancer.

## 1. Introduction

Breast cancer is a malignant tumor originating in the epithelial cells of the breast, characterized by abnormal cell proliferation and invasion of surrounding tissues. Breast cancer ranks among the most common cancers in women worldwide and is the leading cause of cancer-related deaths in women. The incidence and mortality rates of breast cancer are increasing, impacting countries at different stages of development. In 2018, approximately 1.4 million new breast cancer cases were reported worldwide, leading to over 490,000 deaths, with a significant impact on developing countries.^[1]^ According to the 2020 GLOBOCAN estimates from the International Agency for Research on Cancer, breast cancer surpassed lung cancer as the most prevalent cancer among women, with approximately 2.3 million new cases (11.7%) and 685,000 deaths.^[2]^ Mortality rates for breast and cervical cancers are significantly higher in women from developing countries compared to those in developed countries.^[3]^ In developed countries, such as the United States, breast cancer affects approximately 12.38% of women,^[4]^ yet a decline in both incidence and mortality has been observed.^[5]^ In South America, Africa, and Asia, breast cancer incidence is rising, attributed to limited access to advanced diagnostics and treatment.^[6]^ In China, breast cancer has the highest incidence and mortality rates among female malignancies, with a mortality rate of around 6.9%.^[7]^ The lack of early screening, diagnostic methods, and affordable treatment options further exacerbates the global health burden of breast cancer.^[8]^

International guidelines recommend breast cancer treatment strategies that include chemotherapy, radiation, targeted therapy, immunotherapy, and hormone therapy administered pre- and post-surgery.^[9]^ The primary goal is to improve patients’ quality of life and survival rates. Additionally, certain plants containing natural compounds may present novel treatment options within traditional Chinese medicine (TCM).^[10]^ The National Comprehensive Cancer Network in the United States recommends meditation, massage, yoga, tai chi, and acupuncture for alleviating cancer-related symptoms, many of which are consistent with TCM practices.^[11]^ After thousands of years of development, TCM offers effective breast cancer treatments, particularly through herbal medicine and massage, and is endorsed as a recommended approach by the State Administration of Traditional Chinese Medicine. TCM posits that blood stasis plays a critical role in tumor development. Recent clinical studies further suggest that breast cancer patients often exhibit a hypercoagulable state.^[12]^ TCM therapies aimed at enhancing blood circulation and resolving blood stasis can improve microcirculation, thereby alleviating hypoxic conditions in tumor tissues. In vitro studies indicate that these TCM treatments can inhibit the proliferation and spread of HER-2-positive breast cancer cells.^[13]^ However, patients may experience long-term complications arising from multiple factors. Research has shown that moxibustion can improve blood and lymph circulation, boost immune function, and enhance chemotherapy effectiveness,^[14]^ thereby reducing patients’ Visual Analog Scale scores.^[15]^

To enhance cancer treatments and survival rates, comprehensive research is essential to fully leverage TCM methods. Although bibliometric analysis has been applied to several fields within gynecology, few studies have focused specifically on TCM in breast cancer, and the distribution of literature in this field remains relatively scattered. This fragmentation limits medical practitioners’ ability to gain a comprehensive and intuitive understanding of the current trends and research status on TCM treatment for breast cancer. Bibliometrics is an interdisciplinary field that employs mathematical and statistical techniques to quantitatively analyze diverse forms of knowledge. Applied bibliometrics enables the assessment of a discipline’s social and scientific significance over a specific period. Bibliometrics allows scholars to quantitatively identify research hotspots and development trends, thereby enabling scientific and informed academic decision-making. This article analyzes the current research status in the field of Chinese medicine for breast cancer using bibliometric methods and identifies future directions for research in this area.^[16]^ Bibliometric analysis tackles this challenge by examining contributions across countries, authors, journals, and other factors within comparative literature.^[17]^

From a theoretical perspective, this approach fosters the integration of cross-cultural medical concepts, allowing the global medical community to understand the holistic framework and diagnostic principles of Chinese medicine in breast cancer treatment, thereby facilitating the fusion of diverse medical theories. Concurrently, it supports evidence-based practice by quantitatively evaluating Chinese medicine research outcomes, providing both domestic and international clinicians with evidence-based insights into its effectiveness and safety in treating breast cancer. From a technological application standpoint, this analysis can optimize treatment combinations, enabling international clinicians to adapt Chinese medicine techniques based on analytic outcomes and local patient characteristics. Additionally, it enhances the efficiency of medical resource allocation, allowing resources to be distributed effectively in accordance with Chinese medicine research findings at different stages of breast cancer. For international research cooperation, this analysis can guide the direction of collaborative research by identifying research hotspots and emerging issues, thereby supporting global partnerships. It also advances international academic exchange and talent development by systematically reviewing literature to identify research gaps, facilitating academic exchange and collaborative training, and injecting new vigor into global clinical practice.^[18]^

Recently, the combination of bibliometric analysis and visualization methods has become prevalent in biological research.^[19]^ This paper aims to analyze the current state and trends in TCM-based treatments for breast cancer through bibliometric methods. The goal is to provide insights into global research progress and suggest more effective therapeutic approaches.^[20–22]^ This study employs CiteSpace, VOSviewer, and SCImago to conduct a visual analysis of literature on TCM treatment for breast cancer from the Web of Science, intending to establish a foundation for future research.

## 2. Data analysis

### 2.1. Ethical statement

The study does not involve animals or human subjects, and therefore, ethical approval is not required.

### 2.2. Data sources and retrieval strategies

**Figure 1.**
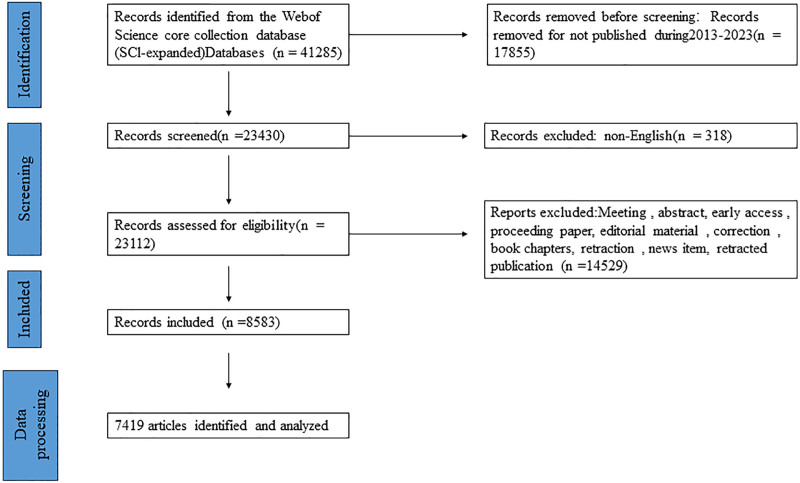
Flowchart of the inclusion and exclusion of publication.

A systematic and comprehensive search was conducted to retrieve literature on traditional Chinese medicine treatments for breast cancer. A systematic and comprehensive search was conducted to retrieve literature on traditional Chinese medicine treatments for breast cancer. The search timeframe covered the years from 2013 to 2023. The search strategy was defined as follows: TS = (“Female breast cancer” OR “breast cancer”) AND TS = (“traditional Chinese medicine” OR “acupuncture” OR “massage” OR “moxibustion”). The search was limited to English-language literature, focusing on clinical trials and review articles. Relevant data, such as titles, keywords, authors, countries, publication years, journals, citations, and institutions, were independently extracted from all eligible publications. A flowchart summarizing this analytical process is presented in Figure 1.

### 2.3. Research methods and visualization

Publications from Web of Science were imported into VOSviewer (version 1.6.11), developed by Leiden University in the Netherlands, and CiteSpace (version 5.8.R2), developed by Drexel University in Philadelphia, PA, to conduct co-authorship analysis across countries, journals, and institutions. Additionally, keyword co-occurrence analysis was performed. Statistical evaluation of the number of studies published by each country was performed using Microsoft Excel 2019. Leiden University’s Centre for Science and Technology Studies provides various products based on VOSviewer. CiteSpace is an information visualization software developed by Professor Chaomei Chen using the Java programming language. Institutional, country, and keyword highlight analyses were conducted using CiteSpace. Keyword co-occurrence analysis was used to identify prominent topics and investigate research frontiers in this field. These maps illustrate knowledge structures, patterns, and distributions in scientific literature. This tool assists researchers in navigating the knowledge base, identifying research hotspots, and exploring frontiers within specific research fields.^[23]^ Studies indicate that CiteSpace has become an influential analytical tool in bibliometrics.^[24–26]^

WoSCC includes “full records and cited references” in the literature selection, allowing export in plain text format. Downloaded files were renamed as “download_x.” CiteSpace was run to import text files, and duplicate files were removed, resulting in 0 duplicates and 7419 valid documents. The analysis period (time slices) was set from 2013 to 2023, with each year as a single time slice. Node types included authors, countries, institutions, keywords, and references. These nodes were used to generate network knowledge maps, including author cooperation, country cooperation, institution cooperation, keyword co-occurrence, and literature co-citation. Selection criteria were set to include the top N = 10; pruning was applied as follows: pathfinder selection, partial network switching (pruning time slice network), and pruning merged networks to obtain clearer, more prominent network maps.

## 3. Results

### 3.1. Basic statistical analysis

**Figure 2.**
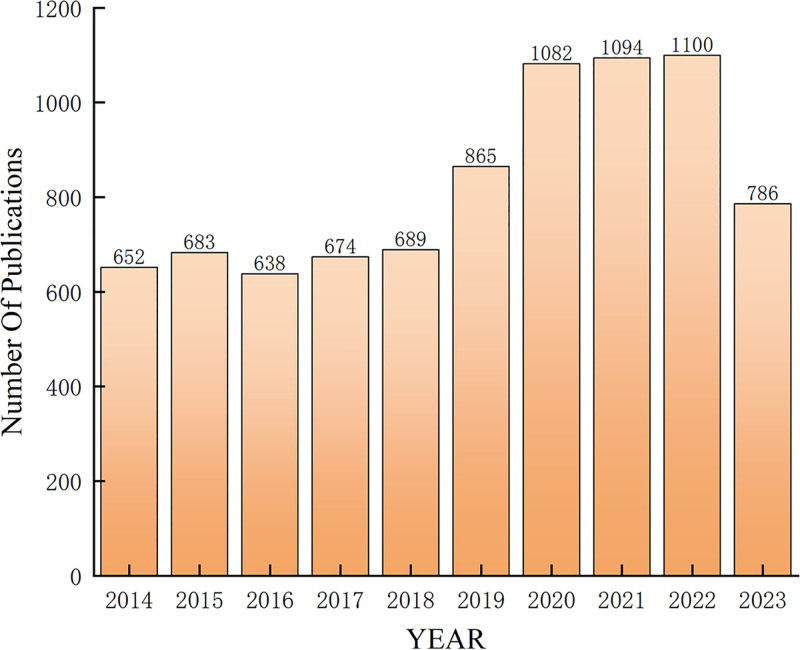
Annual trends in publications and citations in the field of traditional Chinese medicine for breast cancer, 2013 to 2023.

A total of 7419 articles were analyzed using a bibliometric retrieval strategy spanning an 11-year period from January 2013 to 2023. Research on TCM for breast cancer treatment displays a dual-phase development trend. The first phase, from 2013 to 2018, was relatively stable, averaging over 600 articles published annually. The second phase, from 2019 to 2023, saw annual publications exceed 800 articles, peaking at 1100 in 2022. The increasing publication count reflects growing global interest in TCM for breast cancer treatment, with marked increases in both articles and citations.^[27]^ The period from 2020 to 2022 was especially productive, with high publication volumes, though there was a slight decline in 2023 (Fig. 2).

### 3.2. Analyzing the most productive countries and international cooperation

**Figure 3.**
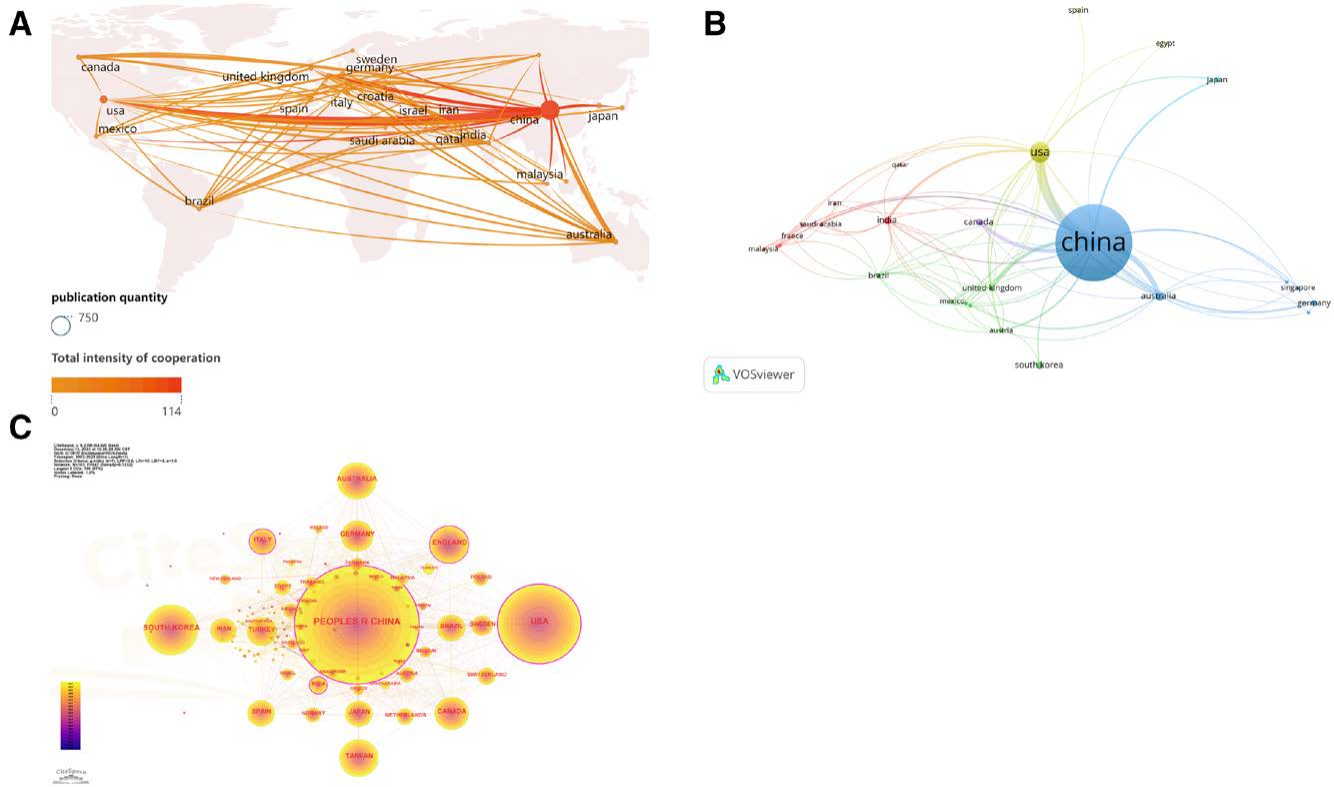
Geographical distribution and networks of traditional Chinese medicine in treating breast cancer at the national regional level. (A) Map based on the geographical distribution of total publications in different countries, different nodes represent different countries, and the size of the nodes represents the number of publications. (B) VOS viewer network map of related countries and regions, different nodes represent the number of communications from different countries, and the thickness of the connecting lines represents the closeness of the relationship between countries; (C) network map of relevant countries and regions. The size of the circle represents the number of national publications, while the pink circumference indicates that its research represents the development of research hotspots.

The 7419 selected articles represent 102 countries and regions. China leads with 3201 articles (43.14%), followed by the United States with 1464 articles (19.73%) and South Korea with 630 articles (8.49%). The top 10 countries also include Iran, the United Kingdom, Australia, Canada, Germany, Turkey, and Brazil (Table 1). Figure 3A presents a geographic map illustrating the relationship between publication counts and countries. Different nodes represent different countries and the size of the node represents the number of publications. The thickest connecting line appears between China and the United States, indicating the strongest relationship and closest cooperation between these 2 countries. Additionally, China maintains stronger ties with Australia and South Korea than with other countries, except for the United States, suggesting closer cooperation with China. The collaboration network was visualized using VOS viewer (Fig. 3B), where each node represents a country, and node size reflects centrality (centrality ≥ 0.1). Different nodes represent the amount of communication between different countries, and the thickness of the connecting lines represents the closeness of the relationship between countries. Larger nodes represent countries with more influential citations.

**Table 1:** Top 10 countries in published research on TCM for breast cancer treatment from 2013 to 2023.

Rank	Count	Country	Citations	Average article citations	H-index
1	3201	PEOPLES R CHINA	50,630	14.66	74
2	1464	USA	49,938	26.44	87
3	630	SOUTH KOREA	10,994	13.59	46
4	346	ENGLAND	19,471	41.34	64
5	337	AUSTRALIA	41,912	36.37	53
6	264	CANADA	14,714	42.98	51
7	250	GERMANY	8704	28.08	44
8	203	TURKEY	5850	19.83	33
9	189	BRAZIL	4012	15.55	33
10	178	IRAN	3805	15.40	32

TCM = traditional Chinese medicine.

Meanwhile, the thickness of the linkage lines between countries indicates the strength of their connections, with thicker lines between China, the United States, Australia, and Canada, signifying closer connections. The circle sizes in Figure 3C represent the number of publications, with larger circles indicating higher publication counts. These countries exhibit active collaboration. China, the United States, and South Korea lead as the top 3 countries in publication output. CiteSpace was used to measure the centrality of each country/region, reflecting their importance within the network. The United States ranked highest in centrality with a score of 0.37, surpassing China’s 0.22, underscoring the U.S.’s greater influence in this research domain. At the same time, the pink color surrounding the circles indicates the prominence of research hotspots in these countries, highlighting China and the United States as leaders in advancing traditional Chinese medicine for breast cancer treatment on the global stage.

### 3.3. Analysis of the most productive institutions in TCM treatment of breast cancer

**Figure 4.**
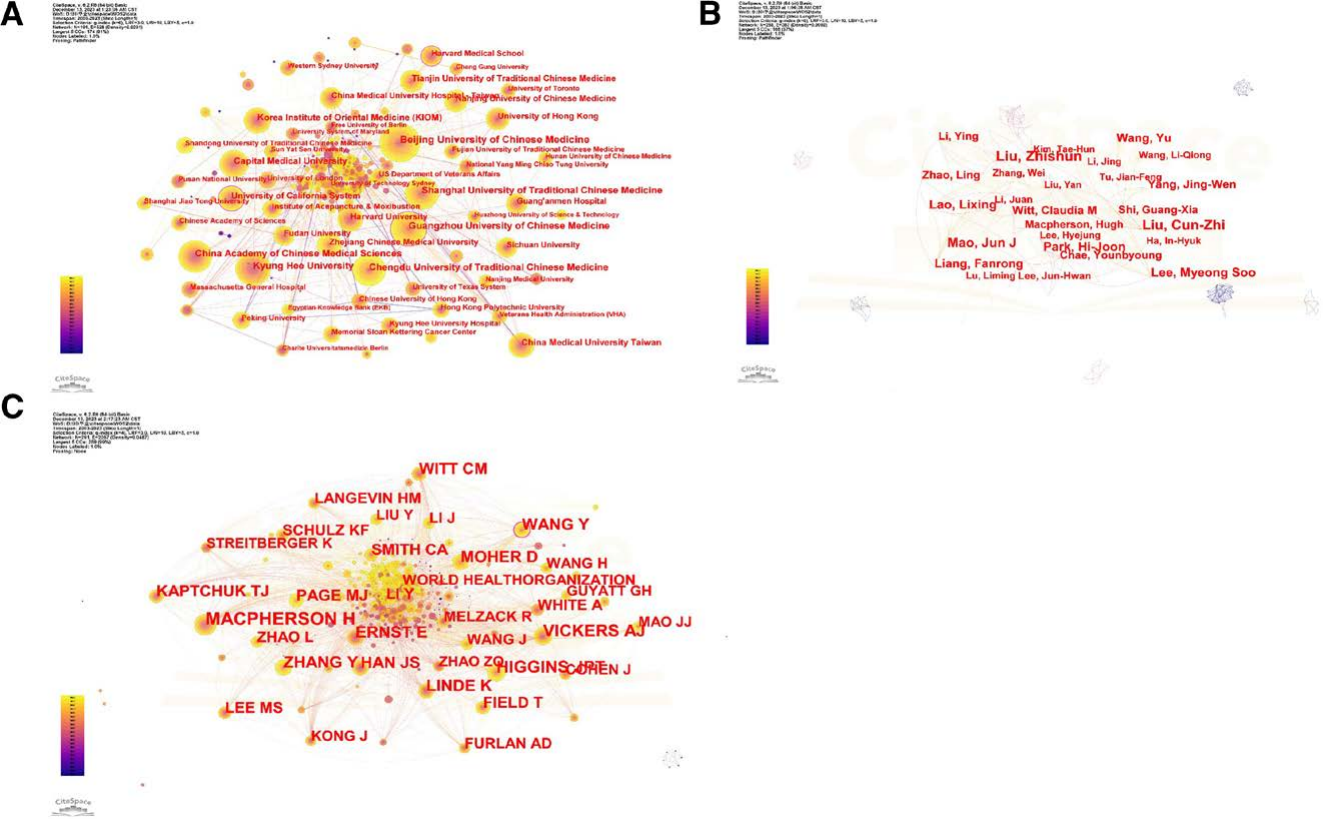
The visualization map of institutions, authors, and coauthors related to TCM research on breast cancer from 2013 to 2023. (A) 2013 to 2023 Chinese medicine treatment of breast cancer research related institutions visualization map, different nodes represent different organizations, and the size of the node represents the number of messages sent by each organization; (B) visualization of authors of studies related to TCM treatment of breast cancer from 2013 to 2023.Different nodes represent different authors, and different letter sizes represent the number of posts by that author; (C) co-citation of authors occurs, each node represents a co-cited author, and denser lines connected to it indicate more citations. TCM = traditional Chinese medicine.

From the 191 institutions involved in publications, only those with at least 50 published articles were included in the analysis (Fig. 4A). Each node in the graph represents an organization, with node size indicating the number of articles published by each institution. A total of 23 institutions were selected for further analysis. Among these, the top 5 institutions by publication count were Beijing University of Chinese Medicine (359 articles), Guangzhou University of Chinese Medicine (268 articles), Kyung Hee University (268 articles), Shanghai University of Traditional Chinese Medicine (242 articles), and Chengdu University of Traditional Chinese Medicine (241 articles). Notably, the number of publications did not always directly correlate with citation frequency. The centrality scores were 0.13 for Guangzhou University of Chinese Medicine and 0.08 for both Beijing University of Chinese Medicine and Kyung Hee University, indicating their influence within the collaboration network. Among the top institutions, Guangzhou University of Chinese Medicine demonstrated the highest influence, as indicated by its centrality score (Table 2).

**Table 2:** Top 10 institutions with the highest number of publications and average citations.

Rank	Count	Centrality	Institutions	Citations	Average article citations	H-index
1	359	0.08	Beijing University of Chinese Medicine	7055	17.12	37
2	268	0.13	Guangzhou University of Chinese Medicine	5068	15.45	35
3	268	0.08	Kyung Hee University	3895	12.56	28
4	242	0.02	Shanghai University of Traditional Chinese Medicine	3354	11.89	28
5	241	0.05	Chengdu University of Traditional Chinese Medicine	5300	18.86	33
6	235	0.05	China Academy of Chinese Medical Sciences	4477	16.22	34
7	180	0.01	Capital Medical University	5098	19.17	33
8	158	0.02	Korea Institute of Oriental Medicine	4089	19.66	34
9	155	0.05	China Medical University Taiwan	2983	15.87	31
10	144	0.15	University of California System	5325	29.1	31

### 3.4. Analysis of high impact authors of TCM treatment of breast cancer

A total of 33,354 authors have contributed to research on TCM for breast cancer. In the graph, each node represents an author, with node size indicating the author’s publication count.

Core authors were identified using Price’s Law, where *M* is the minimum number of publications, with *M* ≤ 0.749 (Nmax 1/2), and Nmax represents the total number of publications by the leading author in the field. Cite Space analysis revealed that Liu Zhishun has the highest number of publications (52 articles), with an *M* value of 19.47. Authors with more than 19 publications are considered core contributors to the field. This study identified 14 core authors (Table 3). Additionally, 16 authors have published at least 10 papers, and 99 authors have published at least 5 papers (Fig. 4B). Co-cited author analysis examines pairs of authors whose works are cited together by a third author, with higher co-citation frequencies indicating closer academic alignment and greater research density. Each node in the graph represents a co-cited author, with denser lines connecting nodes indicating higher citation frequencies. The majority of the authors are from China, underscoring the significant contributions and leadership of Chinese scholars in this field. Other contributors include researchers from South Korea, the United States, and other nations. The author co-authorship analysis (Fig. 4C) reveals many light-colored nodes, indicating an increasing number of researchers involved. Co-citation frequency and centrality analysis showed that Y MacPherson H had the highest citation count (567), while Wang Y had the highest centrality score (0.15), highlighting their significant impact in the field.

**Table 3:** Top 10 authors and co-cited authors related to TCM for breast cancer treatment for 2013 to 2023.

Rank	Total publications	Centrality	Author	Citations	Average article citations	H-index
1	52	0.05	Liu, Zhishun	1641	22.79	26
2	50	0.06	Liu, Cun-Zhi	1586	23.32	20
3	40	0.10	Mao, Jun J	1686	29.58	22
4	37	0.22	Lao, Lixing	2020	31.08	23
5	37	0.30	Liang, Fanrong	1413	27.17	19
6	33	0.07	Yang, Jing-Wen	1078	21.14	19
7	27	0.08	Lee, Myeong Soo	1017	20.76	18
8	26	0.07	Shi, Guang-Xia	1123	23.41	20
9	25	0.03	Park, Hi-Joon	1066	24.23	18
10	25	0.10	Li, Ying	1018	26.10	15

TCM = traditional Chinese medicine.

### 3.5. Analysis of high impact journals of TCM treatment of breast cancer

**Figure 5.**
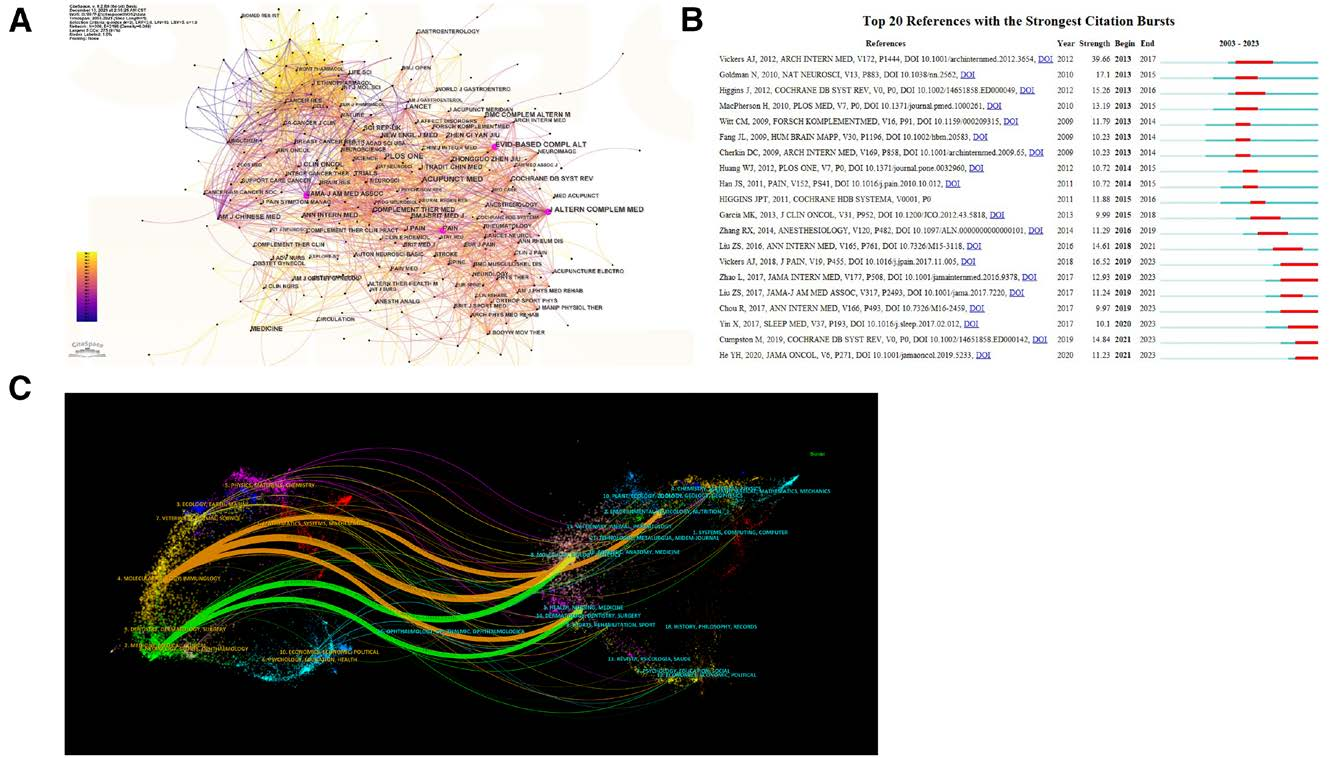
Literature analysis of TCM treatment of breast cancer related publications. (A) Analysis map of co-cited literature in the past 10 years. Larger nodes indicate a higher frequency of being referenced; (B) the top 20 references with the strongest frequency bursts. The green bar indicates the time period in which the keyword appears; the red bar chart represents the interval between keyword bursts found, indicating the year the outbreak started, the year it ended, and the duration. (C) 2013 to 2023 Chinese medicine treatment of breast cancer journals and research fields double map overlay visualization. The ovals in the figure represent the number of publications corresponding to the journal. The longer the horizontal axis of the ellipse, the more authors it represents; the longer the vertical axis of the ellipse, the more papers published in the journal. TCM = traditional Chinese medicine.

Among the 7419 articles on TCM for breast cancer, a total of 1325 journals were involved. The journals with the highest publication frequencies were *Evidence-Based Complementary and Alternative Medicine* (2591 articles, 34.91%), *Journal of Alternative and Complementary Medicine* (2087 articles, 28.13%), and *PLOS ONE* (2005 articles, 27.02%) (Table 4); their average citation counts were 6.65, 15.75, and 26.64, with respective H-indexes of 27, 28, and 42. These journals span a range of topics, including advanced fields like herbal medicine, acupuncture, massage, and moxibustion, as well as neurology, integrative medicine, and complementary medicine. Additional details of other major journals are shown in Figure 5A. Among these, the journals *Evidence-Based Complementary and Alternative Medicine*, *Journal of Alternative and Complementary Medicine*, *PAIN*, and *JAMA* – *Journal of the American Medical Association* show close intercitation relationships. In the graph, larger nodes represent more frequent citations, indicating higher significance of the articles. Larger nodes in the graph indicate more frequent citations and higher importance of the article.

**Table 4:** Top 10 journals and co-cited journals that published articles on TCM for breast cancer treatment for 2013 to 2023.

Rank	Count	Centrality	Journals	Citations	Average article citations	H-index
1	2591	0.13	EVID-BASED COMPL ALT	3562	6.65	27
2	2087	0.15	J ALTERN COMPLEM MED	3212	15.75	28
3	2005	0.08	PLOS ONE	5861	26.64	42
4	1856	0.05	ACUPUNCT MED	5895	11.56	32
5	1803	0.09	COCHRANE DB SYST REV	7458	54.44	51
6	1648	0.06	LANCET	611	23.5	14
7	1577	0.16	PAIN	913	26.09	18
8	1521	0.04	BMJ-BRIT MED J	378	31.5	8
9	1490	0.13	JAMA-J AM MED ASSOC	1905	59.53	10
10	1405	0.07	COMPLEMENT THER MED	49	2.45	4

TCM = traditional Chinese medicine.

### 3.6. Co-citation reference clusters and reference burst

Co-citation refers to 2 papers being cited together by a third paper, where stronger co-citation suggests higher burst strength, indicating greater influence within the field. The analysis of co-cited references identified the top 20 references with the highest burst rates (Fig. 5B), representing the most influential works in recent research on TCM for breast cancer and highlighting research trends across different periods. Among these, Page MJ (2021, *BMJ – British Medical Journal*) holds the highest citation count with 239 citations. Citation burst analysis reveals that the top 3 references in burst strength are Vickers AJ (2012, *Archives of Internal Medicine*, burst strength = 39.66), Goldman N (2010, *Nature Neuroscience*, burst strength = 17.1), and Vickers AJ (2018, *Journal of Pain*, burst strength = 16.52). Each of these highly cited, central references addresses different aspects of supportive care in breast cancer treatment. Greenlee H et al conducted a systematic review of complementary and integrative therapies, finding that music therapy, meditation, and yoga effectively alleviated anxiety, while yoga, massage, and music therapy helped with depression. They also reported that massage and acupuncture reduced chemotherapy-induced nausea and vomiting.^[28]^ Another highly cited source highlights acupuncture’s effectiveness in managing chronic pain, demonstrating its validity as a supportive treatment for breast cancer pain.^[29]^

### 3.7. Journals disciplines distribution

Dual map overlays of journals offer insights into cross-disciplinary distribution, citation pathways, and major research centers involved in TCM for breast cancer treatment. In the dual map overlay analysis (Fig. 5C), citing journals are displayed on the left, while cited journals appear on the right, with colored lines representing citation links. Line thickness indicates connection strength. The analysis reveals that journals on TCM for breast cancer are mainly cited in fields such as molecular biology, immunology, neurology, dermatology, and dentistry, and are frequently referenced by journals in environmental toxicology, nutrition, biology, anatomy, healthcare, nursing, medicine, and pharmacology. Overall, TCM treatment for breast cancer is closely linked to research in basic medicine, nutrition, healthcare, and clinical fields. Future research should focus on further strengthening these interdisciplinary connections and applications.

### 3.8. Keywords

#### 3.8.1. Co-occurrence analysis

**Figure 6.**
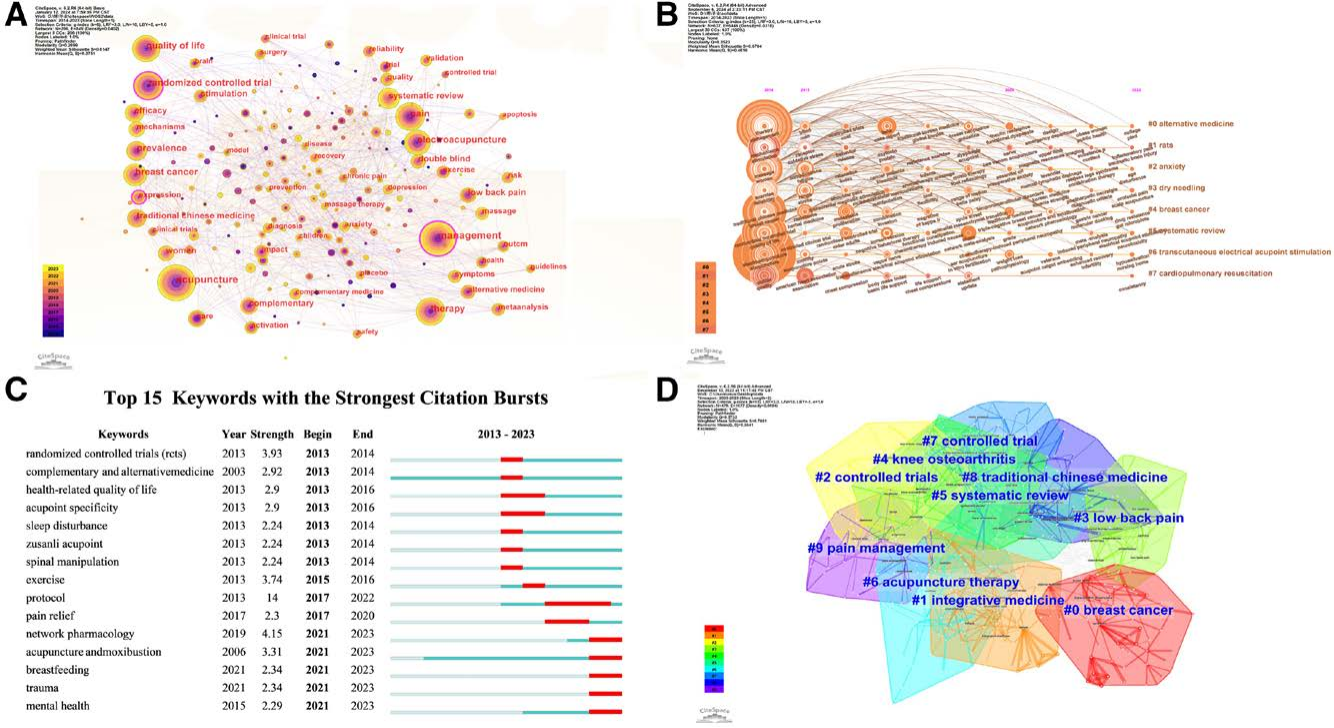
Keyword analysis of publications related to TCM treatment of breast cancer. (A) Network visualization of co-occurrence of keywords related to TCM treatment of breast cancer. Large nodes represent high-frequency keywords; (B) keywords related to Chinese medicine treatment of breast cancer timeline co-occurrence network graphs. The size of the nodes indicates the degree of hotness of the research trend in the corresponding year, and the connecting lines of the nodes indicate the development trend of the hotspots; (C) top 20 keywords with the strongest co-occurrence frequency outbreaks. Represents the time interval of keyword outbreak discovery, each bar represents a year, and the red bars represent strong citation bursts. (D) Cluster graph of high-frequency keywords in relevant literature. Nodes in the diagram represent references, and colored fonts represent cluster labels. Nodes with the same color are located in the same cluster, that is, they belong to the same cluster theme. TCM = traditional Chinese medicine.

Keywords and their network diagrams concisely capture research topics and help pinpoint hotspots in the field. Analysis of keywords in this field generated a knowledge map with multiple nodes and connections, as illustrated in Figure 6A. The modularity *Q* score of 0.5767 and weighted average silhouette value of 0.7981 indicate a clear cluster structure and reliable results. After excluding search-related keywords like “breast cancer” and “traditional Chinese medicine,” “acupuncture” appeared most frequently, with 1031 occurrences and the highest centrality of 0.16. Analysis of high centrality and frequency keywords reveals a primary research focus on breast cancer treatments involving “acupuncture,” “aromatherapy,” and “massage,” with additional keywords such as “therapy,” “pain,” and “quality of life.”

#### 3.8.2. Clustering analysis

The LST algorithm was applied to conduct clustering analysis on keywords, yielding a silhouette value of 0.57, which confirms the clustering results’ reliability. A total of 10 clusters were identified and labeled as follows: #0 breast cancer, #1 integrative medicine, #2 controlled trials, #3 low back pain, #4 knee osteoarthritis, #5 systematic review, #6 acupuncture therapy, #7 controlled trial, #8 traditional Chinese medicine, and #9 pain management (Fig. 6D). Table 5 provides details of each cluster. Notably, “acupuncture” serves as a prominent complementary therapy for breast cancer treatment. As shown in Figure 6B, the node size reflects the prominence of research trends in each year, while connecting lines indicate the evolution of research hotspots over time. Eight clusters were generated, with #3 dry needling and #6 transcutaneous electrical acupoint stimulation as the most concentrated keywords. This indicates a research focus on acupuncture-related topics during this period, highlighting their importance in TCM-based breast cancer treatments. Over time, the research focus shifted towards #0 alternative medicine and #5 systematic review, both gaining greater emphasis in the mid-term. Additional research topics during this period included #1 rats, #2 anxiety, #4 breast cancer, and #7 cardiopulmonary resuscitation.

**Table 5:** Top 10 high-frequency keywords in literature related to TCM for breast cancer treatment from 2013 to 2023.

Rank	Count	Total link strength	Keywords
1	2122	2640	Acupuncture
2	505	1062	Network meta-analysis
3	453	843	Randomized controlled trial
4	422	905	Quality of life
5	403	584	Massage therapy
6	371	548	Complementary and alternative medicine
7	363	339	Breast cancer
8	275	484	Pain
9	221	320	Traditional Chinese medicine
10	2122	2640	Efficacy

TCM = traditional Chinese medicine.

#### 3.8.3. Keyword burst analysis

Bursting keywords are those that experience a sharp increase in frequency within a particular period, revealing trends in a research field. A burst analysis of keywords produced a map that ranks the top 22 keywords by burst strength (Fig. 6C). In the graph, each line represents a year, with red bands indicating periods of strong citation surges. The analysis identifies 2 main phases in this field: the first phase, from 2013 to 2016, primarily focused on clinical investigations, particularly on topics like “randomized controlled trials,” “quality of life,” “protocol,” and “chemotherapy-induced peripheral neuropathy” and other related topics. The second phase, from 2017 to 2023, emphasized research in “network pharmacology,” “neuromodulation,” “non-pharmacological interventions mechanism of action,” and “mechanism of action.”

## 4. Discussion

This study examines publication patterns in core literature from the Web of Science, summarizing the trends and research hotspots in TCM treatment for breast cancer over the last decade. A search identified 7419 references, revealing a steady global increase in TCM-related breast cancer articles from 2013 to 2023, underscoring the growing interest in this approach.

### 4.1. Basic situation

In analyzing the national and regional distribution, China, as the birthplace of TCM, stands out in TCM research. However, TCM literature is drawing growing interest from international researchers. Because of geographic proximity and cultural foundations, East Asian countries such as Japan and South Korea are more receptive to TCM research, with high public acceptance levels facilitating clinical research. Additionally, research from the United States, South Korea, and other regions is expanding, with the United States’ involvement deepening, highlighting the need for greater international collaboration in advancing TCM. Developed countries, such as the United States, show substantial interest in Chinese medicine treatments, underscoring that TCM approaches and their effects are gaining acceptance and scholarly attention in Western countries, signaling major advances for TCM development.

The most prominent contributing author was Liu Zhishun, whose research focused on acupuncture for gynecological diseases, such as uterine fibroids and cancer pain management.^[30–34]^ The second most prolific author, Liu Cun-Zhi, explored acupuncture’s effects on the nervous system and topics such as bee acupuncture.^[35–38]^ The most cited author, MacPherson H, investigated neurostimulation, manual techniques, acupuncture, and its therapeutic effects using systematic analysis. These findings indicate that Chinese authors have made substantial contributions to this research field, yet there is a need for enhanced collaboration with authors from other countries.^[39–41]^ The co-citation network of authors is denser than the authorship network itself.^[42]^

The most frequently co-cited author was Vickers AJ, known for his extensive research on gynecological cancers and associated symptoms, including gastrointestinal issues, swelling, and pain experienced during examinations. Among the journals, *Evidence-Based Complementary and Alternative Medicine*, with 2591 articles, and *Journal of Alternative and Complementary Medicine*, with a centrality score of 0.15, were the most influential. In terms of institutional collaboration, Beijing University of Chinese Medicine produced the highest number of publications, whereas Guangzhou University of Chinese Medicine achieved the highest centrality score. In national collaboration, China and the United States led in publication volume, with China producing a larger body of literature. However, the United States had the highest centrality, suggesting a greater research depth and academic influence in this field.

### 4.2. Research hotspots

The analysis of commonly used keywords revealed the research hotspots in TCM treatment for breast cancer, focusing on 3 key treatment modalities: acupuncture, breastfeeding, and TCM therapies. Acupuncture has been utilized for over 2000 years to regulate bodily functions and conditions by stimulating specific points on the body. Complementary therapies in Western countries now incorporate the principles of TCM to address the root causes of disease, contributing to the broader acceptance of acupuncture. Studies have demonstrated the effectiveness of acupuncture in treating cancer and related symptoms, and acupuncture for breast cancer requires further in-depth investigation. The appropriate combination of acupuncture points can enhance treatment effectiveness. One study reported that over 24.1% of breast cancer patients experienced “substantial benefit” or “complete resolution of symptoms,” with an average reduction in symptom severity. The majority of patients experienced immediate (34%) or gradual (40.4%) benefits.^[43]^ Acupuncture reduced elbow diameters, alleviated upper extremity lymphedema, and improved treatment outcomes in breast cancer patients.^[44]^ Acupuncture also benefits gynecologic pelvic surgery.^[45]^ Electroacupuncture, transcutaneous electrical stimulation of acupuncture points, and traditional whole-body acupuncture are effective in managing perioperative pain.^[46]^ Further studies have highlighted the immunomodulatory effects of acupuncture and its long-term efficacy in relieving chronic pain.^[40]^ Current statistics suggest that acupuncture is generally a safe treatment, although minor adverse events may occur.^[47]^ The aforementioned studies demonstrate that the clinical application of acupuncture in the treatment of breast cancer can effectively relieve pain, reduce edema, and improve the quality of patients’ survival. Continued research in the field of acupuncture for breast cancer-related symptoms is necessary to refine treatment strategies and enhance the efficacy of breast cancer treatment.

Breastfeeding is the primary method of infant rearing, which significantly impacts both infant health and maternal well-being. Modern research indicates that breastfeeding reduces the risk of breast cancer in mothers.^[48]^ The 4th edition of the European Cancer Code advises that breastfeeding reduces the risk of cancer in mothers. Breastfeeding for an additional 5 months is estimated to reduce the risk of breast cancer by 5%. The longer women breastfeed, the lower their risk of developing breast cancer.^[49]^ Studies have shown that the prevalence of breast cancer is 1.37 times higher among women who do not breastfeed compared to those who breastfeed.^[50]^ A review of medical records from 524 breast cancer patients revealed that maternal lactation for more than 3 months was a statistically significant factor in diagnosing fewer breast cancer cases in non-morbidly obese women. Therefore, maternal lactation may offer protective effects against breast cancer.^[51]^ Among menstruating women aged 25 years and older, 1712 new breast cancer cases (3.2% of all new cases) occurred among those who breastfed for <6 months, while breastfeeding practices were associated with the prevention of 765 breast cancer cases.^[52]^ The duration of breastfeeding is inversely associated with the likelihood of developing breast cancer, with a 4.3% reduction in relative risk for every 12 months of breastfeeding. It has been estimated that if the average number of births and the duration of lifetime breastfeeding in women were to match the levels prevalent in the developing world until recently, the cumulative incidence of breast cancer in developed countries would be reduced by more than half by age 70, from 6.3 to 2.7 per 100 women.

Breastfeeding may account for approximately two-thirds of the estimated reduction in breast cancer incidence.^[53]^ Pregnancy and breastfeeding are the most effective protective factors against breast cancer, with a woman’s risk decreasing in proportion to both the duration of breastfeeding and the number of full-term pregnancies.^[54]^ This suggests that breastfeeding as a preventive measure against breast cancer is an emerging trend in research, which may alleviate patient suffering and improve therapeutic strategies. Breastfeeding has a protective effect on mothers, with longer breastfeeding durations associated with a reduced risk of breast cancer. This aligns with the traditional Chinese medicine approach of preventing disease before its onset. Building upon this research, further systematic clinical studies could help lower breast cancer rates and reduce the number of individuals affected by the disease.

TCM provides a valuable alternative for breast cancer treatment with fewer side effects. TCM can inhibit tumor growth, enhance treatment efficacy, and improve patients’ quality of life, particularly for those recovering from chemotherapy.^[55]^ TCM is effective across various breast cancer subtypes, with meta-analyses showing improvement in short-term outcomes (*Z* = 7.05) and extended survival rates with early treatment.^[56]^ TCM complements modern medicine in postoperative breast cancer care, significantly influencing bone metastasis treatment (RR = 1.10, *P* < .05) and providing notable pain relief.^[57,58]^ These findings demonstrate the effectiveness of TCM in reducing adverse reactions.^[59]^ Research indicates that 48.90% of patients utilized TCM during treatment, with 64 cases (95.50%) reporting improvements in symptoms such as better sleep, reduced fatigue, and enhanced physical and mental well-being.^[60]^ Patients who received TCM treatment reported a significantly higher quality of life than those who did not.^[59]^ As TCM evolves, growing evidence underscores its significant antitumor activity. TCM plays a crucial role throughout the entire cancer treatment process, including postoperative recovery, radiotherapy, and chemotherapy, rather than only in advanced stages. Certain TCM treatments, such as Liu Jun Zi Tang, coumarin, and escin, have been shown to alleviate postoperative symptoms, including fatigue, pain, appetite loss, diarrhea, nausea, vomiting, and edema. Combining TCMs such as ginseng, astragalus, ban zhi lian, Shenqi Fuzheng Injection, and Kanglaite Injection with radiotherapy and chemotherapy can enhance treatment efficacy while reducing side effects and complications.^[61]^ Astragaloside IV, the primary active component of astragalus, possesses anti-inflammatory and anticancer properties. It can induce cell cycle arrest, apoptosis, and autophagy, while inhibiting cancer cell proliferation, invasion, and metastasis, thereby contributing to the prevention of breast cancer.^[62,63]^ TCM, a widely used complementary therapy in cancer treatment, employs natural compounds and formulas to regulate gene and protein expression through pathways such as PI3K/AKT, JAK2/STAT3, MAPK, and Wnt/β-catenin. These compounds can inhibit the growth and spread of HR+ breast cancer tumors and cooperate with endocrine drugs to overcome resistance.^[64]^ In conclusion, TCM can inhibit tumor growth, reduce surgical complications, and mitigate damage caused by surgery, chemotherapy, or radiation in breast cancer treatment. It is also effective in relieving symptoms such as lymphedema, fatigue, and pain.^[65]^ TCM provides valuable insights for the development of more effective antitumor drugs. Increasing research indicates that the active ingredients in traditional Chinese medicines and formulas play a crucial role in breast cancer treatment and in maintaining overall health. Research on the modulation of traditional Chinese medicine ingredients in pathway-regulating proteins suggests that breast cancer treatment with TCM is increasingly accepted by both patients and doctors. Continued research may uncover more effective and safer treatment modalities. Keyword network analysis demonstrates that TCM is garnering increasing attention, underscoring the growing recognition of its therapeutic value.

### 4.3. Limitations

This study offers a more comprehensive and detailed review of the TCM literature on breast cancer treatment compared to conventional reviews. However, it is important to acknowledge some limitations of this research. Firstly, in studies addressing the treatment of breast cancer with Chinese medicine, bias may arise if the journals involved tend to favor complementary medical approaches over Chinese medicine itself. A shift in research focus may overlook the TCM system of evidence-based treatment, leading to an imbalance between the holistic and localized aspects, and failing to fully reflect disease treatment from the TCM perspective. There is also the potential for misleading research findings to overestimate the effectiveness of complementary medicine approaches, leading to uneven research quality, misleading readers’ judgment of TCM’s scientific value in treating the disease, and impacting subsequent research and clinical practice. Secondly, the software employed may introduce biases and discrepancies between visual representations and actual data, which could affect the results. Moreover, high citation counts do not necessarily indicate high scientific quality. Future efforts should focus on improving data accuracy to enable more precise interpretations of the research landscape. Despite these limitations, the study provides valuable insights into research trends and key areas in TCM treatment for breast cancer. Addressing these limitations in future research will enhance and refine the analysis.

## 5. Future outlook

In conclusion, this study analyzed the global trends in TCM treatment for breast cancer from 2013 to 2023, identifying research hotspots and key findings. However, certain gaps remain: although the research base is established, the need for high-quality studies and reliable evidence has not been fully met, and greater depth is needed to clarify the role of TCM in breast cancer treatment. Although the steady rise in publications indicates international attention and an evolving role for TCM, there is a lack of detailed analysis on the transition from traditional applications to novel uses and the accumulation of new evidence. Additionally, the exploration of new potential indications is inadequately supported by relevant basic science analyses and clinical studies.

Specific recommendations for future research include: strengthening international collaboration, which can be facilitated through academic exchanges and partnerships with leading research institutions; conducting large-scale trials and multicenter studies to comprehensively assess the efficacy of TCM in breast cancer treatment; and undertaking further in-depth studies on specific mechanisms of action. These efforts will advance our understanding and application of TCM in breast cancer treatment and ultimately improve patients’ quality of life and survival rates.

## Acknowledgments

The authors also thank Dr Tie Li and Dr Nan Zhang for their effort in polishing the English content of this manuscript.

## Author contributions

**Conceptualization:** Jiapeng Chai.

**Data curation:** Nan Zhang, Xuefeng Li, Heran Wang, Jiaxun Zhang, Lin Wang, Qi Zhang, Yuxin Jiang.

**Formal analysis:** Tie Li, Jinying Zhao.

**Funding acquisition:** Fuchun Wang.

**Investigation:** Hailin Jiang, Jinying Zhao.

**Methodology:** Tie Li.

**Project administration:** Nan Zhang.

**Resources:** Tie Li.

**Software:** Hailin Jiang, Xuefeng Li, Heran Wang.

**Visualization:** Jiapeng Chai.

**Writing – original draft:** Jiapeng Chai.

**Writing – review & editing:** Fuchun Wang.

## References

[1] Heer E Harper A Escandor N Sung H McCormack V Fidler-Benaoudia MM. Global burden and trends in premenopausal and postmenopausal breast cancer: a population-based study. Lancet Glob Health. 2020;8:e1027–37. 32710860 10.1016/S2214-109X(20)30215-1

[2] Bray F Ferlay J Soerjomataram I Siegel RL Torre LA Jemal A. Global cancer statistics 2018: GLOBOCAN estimates of incidence and mortality worldwide for 36 cancers in 185 countries. CA Cancer J Clin. 2018;68:394–424. 30207593 10.3322/caac.21492

[3] Sung H Ferlay J Siegel RL. Global cancer statistics 2020: GLOBOCAN estimates of incidence and mortality worldwide for 36 cancers in 185 countries. CA Cancer J Clin. 2021;71:209–49. 33538338 10.3322/caac.21660

[4] Siegel R Naishadham D Jemal A. Cancer statistics, 2013. CA Cancer J Clin. 2013;63:11–30. 23335087 10.3322/caac.21166

[5] Lei S Zheng R Zhang S. Global patterns of breast cancer incidence and mortality: a population-based cancer registry data analysis from 2000 to 2020. Cancer Commun (Lond). 2021;41:1183–94. 34399040 10.1002/cac2.12207 PMC8626596

[6] Malvezzi M Carioli G Bertuccio P. European cancer mortality predictions for the year 2016 with a focus on leukemias. Ann Oncol. 2016;27:725–31. 26812903 10.1093/annonc/mdw022

[7] Ginsburg O Bray F Coleman MP. The global burden of women’s cancers: a grand challenge in global health. Lancet. 2017;389:847–60. 27814965 10.1016/S0140-6736(16)31392-7 PMC6191029

[8] DeSantis CE Ma J Gaudet MM. Breast cancer statistics, 2019. CA Cancer J Clin. 2019;69:438–51. 31577379 10.3322/caac.21583

[9] Cardoso F Paluch-Shimon S Senkus E. 5th ESO-ESMO international consensus guidelines for advanced breast cancer (ABC 5). Ann Oncol. 2020;31:1623–49. 32979513 10.1016/j.annonc.2020.09.010 PMC7510449

[10] Ferlay J Soerjomataram I Dikshit R. Cancer incidence and mortality worldwide: sources, methods and major patterns in GLOBOCAN 2012. Int J Cancer. 2015;136:E359–386. 25220842 10.1002/ijc.29210

[11] Lau CHY Wu X Chung VCH. Acupuncture and related therapies for symptom management in palliative cancer care: systematic review and meta-analysis. Medicine (Baltimore). 2016;95:e2901. 26945382 10.1097/MD.0000000000002901 PMC4782866

[12] Zhai B Zhang N Han X. Molecular targets of beta-elemene, a herbal extract used in traditional Chinese medicine, and its potential role in cancer therapy: a review. Biomed Pharmacother. 2019;114:108812. 30965237 10.1016/j.biopha.2019.108812

[13] Jiang H Li M Du K. Traditional Chinese medicine for adjuvant treatment of breast cancer: Taohong Siwu Decoction. Chin Med. 2021;16:129. 34857023 10.1186/s13020-021-00539-7 PMC8638166

[14] Xue N Fu X Zhu Y Da N Zhang J. Moxibustion enhances chemotherapy of breast cancer by affecting tumor microenvironment. Cancer Manag Res. 2020;12:8015–22. 32943934 10.2147/CMAR.S249797 PMC7481310

[15] Bao T Zhi WI Baser RE. Electro-acupuncture versus battle field auricular acupuncture in breast cancer survivors with chronic musculoskeletal pain: subgroup analysis of a randomized clinical trial. Breast Cancer Res Treat. 2023;202:287–95. 37612534 10.1007/s10549-023-07072-1 PMC11218664

[16] Zhang Y Jin D Duan Y. Bibliometric analysis of renal fibrosis in diabetic kidney disease from 1985 to 2020. Front Public Health. 2022;10:767591. 35186833 10.3389/fpubh.2022.767591 PMC8855938

[17] Qiu P Zhou J Zhang J Dong Y Liu Y. Exosome: the regulator of the immune system in sepsis. Front Pharmacol. 2021;12:671164. 33995102 10.3389/fphar.2021.671164 PMC8113812

[18] Zhang J Yu N Wang B Lv X. Trends in the use of augmented reality, virtual reality, and mixed reality in surgical research: a global bibliometric and visualized analysis. Indian J Surg. 2022;84(suppl 1):52–69. 35228782 10.1007/s12262-021-03243-w PMC8866921

[19] Ding X Yang Z. Knowledge mapping of platform research: a visual analysis using VOS viewer and Cite Space. Electron Commer Res. 2020;22:787–809.

[20] Brown NJ Wilson B Shahrestani S. The 100 most influential pub-lications on medulloblastoma: areas of past, current, and future focus review. World Neurosurg. 2021;146:119–39. 33212273 10.1016/j.wneu.2020.11.038

[21] Deora H Ferini G Garg K Narayanan MDK Umana GE. Evaluating the impact of intraoperative MRI in neuro-oncology by scientometric analysis. Life (Basel). 2022;12:175. 35207463 10.3390/life12020175 PMC8877236

[22] Zhang MM Yang KL Cui YC. Current trends and research topics regarding intestinal organoids: an overview based on bibliometrics. Front Cell Dev Biol. 2021;9:14. 10.3389/fcell.2021.609452 PMC8369504 34414174

[23] Chen C. Cite Space II: detecting and visualizing emerging trends and transient patterns in scientific literature. J Am Soc Inf Sci Technol. 2006;57:359–77.

[24] Liu J Wang L Fang H Wang X Wu L Zhang J. Home-based cardiac rehabilitation: a review of bibliometric studies and visual analysis of Cite Space (2012–2021). Medicine (Baltimore). 2022;101:e31788. 36626492 10.1097/MD.0000000000031788 PMC9750688

[25] Synnestvedt MB Chen C Holmes JH. Cite Space II: visualization and knowledge discovery in bibliographic databases. AMIA Annu Symp Proc. 2005;2005:724–8. 16779135 PMC1560567

[26] Chen C Song M. Visualizing a field of research: a methodology of sys-tematic scientometric reviews. PLoS One. 2019;14:e0223994. 31671124 10.1371/journal.pone.0223994 PMC6822756

[27] Chen C Ibekwe-SanJuan F Hou J. The structure and dynamics of cocitation clusters: a multiple-perspective cocitation analysis. J Am Soc Inf Sci Technol. 2010;61:1386–409.

[28] Greenlee H DuPont-Reyes MJ Balneaves LG. Clinical practice guidelines on the evidence-based use of integrative therapies during and after breast cancer treatment. CA Cancer J Clin. 2017;67:194–232. 28436999 10.3322/caac.21397 PMC5892208

[29] Vickers AJ Cronin AM Maschino AC. Acupuncture for chronic pain: individual patient data meta-analysis. Arch Intern Med. 2012;172:1444–53. 22965186 10.1001/archinternmed.2012.3654 PMC3658605

[30] Zhang Y Peng W Clarke J Liu Z. Acupuncture for uterine fibroids. Cochrane Database Syst Rev. 2010;2010:CD007221. 20091625 10.1002/14651858.CD007221.pub2 PMC11270531

[31] Wang W Liu Y Yang X. Effects of electroacupuncture for opioid-induced constipation in patients with cancer in China: a randomized clinical trial. JAMA Netw Open. 2023;6:e230310. 36811861 10.1001/jamanetworkopen.2023.0310 PMC9947731

[32] Zhang YQ Jiao RM Witt CM. How to design high quality acupuncture trials-a consensus informed by evidence (published correction appears in BMJ. 2022;377:o1046). BMJ. 2022;376:e067476. 35354583 10.1136/bmj-2021-067476 PMC8965655

[33] Sun Y Liu Y Liu B. Efficacy of acupuncture for chronic prostatitis/chronic pelvic pain syndrome: a randomized trial. Ann Intern Med. 2021;174:1357–66. 34399062 10.7326/M21-1814

[34] Liu Z Liu Y Xu H. Effect of electroacupuncture on urinary leakage among women with stress urinary incontinence: a randomized clinical. JAMA. 2017;317:2493–501. 28655016 10.1001/jama.2017.7220 PMC5815072

[35] Liu CZ Chen JD Zhang M. Advances on the acupuncture therapies and neuroplasticity. Evid Based Complement Alternat Med. 2018;2018:7231378. 30647763 10.1155/2018/7231378 PMC6311774

[36] Wang L Yang JW Lin LT. Acupuncture attenuates inflammation in microglia of vascular dementia rats by inhibiting miR-93-mediated TLR4/MyD88/NF-κB signaling pathway. Oxid Med Cell Longev. 2020;2020:8253904. 32850002 10.1155/2020/8253904 PMC7441436

[37] Yang NN Yang JW Ye Y. Electroacupuncture ameliorates intestinal inflammation by activating α7nAChR-mediated JAK2/STAT3 signaling pathway in postoperative ileus. Theranostics. 2021;11:4078–89. 33754049 10.7150/thno.52574 PMC7977469

[38] Zhang S Liu Y Ye Y. Bee venom therapy: potential mechanisms and therapeutic applications. Toxicon. 2018;148:64–73. 29654868 10.1016/j.toxicon.2018.04.012

[39] Woods B Manca A Weatherly H. Cost-effectiveness of adjunct non-pharmacological interventions for osteoarthritis of the knee. PLoS One. 2017;12:e0172749. 28267751 10.1371/journal.pone.0172749 PMC5340388

[40] Vickers AJ Vertosick EA Lewith G. Acupuncture for chronic pain: update of an individual patient data meta-analysis. J Pain. 2018;19:455–74. 29198932 10.1016/j.jpain.2017.11.005 PMC5927830

[41] Armour M Smith CA Wang LQ. Acupuncture for depression: a systematic review and meta-analysis. J Clin Med. 2019;8:1140. 31370200 10.3390/jcm8081140 PMC6722678

[42] Carter J Abu-Rustum NR Saban S. Gynecologic survivorship tool: development, implementation, and symptom outcomes. JCO Clin Cancer Inform. 2022;6:e2100154. 35239413 10.1200/CCI.21.00154 PMC8920469

[43] Zayas J Ruddy KJ Olson JE. Real-world experiences with acupuncture among breast cancer survivors: a cross-sectional survey study. Support Care Cancer. 2020;28:5833–8. 32253604 10.1007/s00520-020-05442-9 PMC7541443

[44] Zhang X Wang X Zhang B Yang S Liu D. Effects of acupuncture on breast cancer-related lymphoedema: a systematic review and meta-analysis of randomised controlled trials (published correction appears in Acupunct Med. 2019; 964528419842774). Acupunct Med. 2019;37:16–24. 30845813 10.1136/acupmed-2018-011668

[45] Shah S Godhardt L Spofford C. Acupuncture and postoperative pain reduction. Curr Pain Headache Rep. 2022;26:453–8. 35482244 10.1007/s11916-022-01048-4

[46] Wang M Liu W Ge J Liu S. The immunomodulatory mechanisms for acupuncture practice. Front Immunol. 2023;14:1147718. 37090714 10.3389/fimmu.2023.1147718 PMC10117649

[47] Li H Schlaeger JM Jang MK. Acupuncture improves multiple treatment-related symptoms in breast cancer survivors: a systematic review and meta-analysis. J Altern Complement Med. 2021;27:1084–97. 34449251 10.1089/acm.2021.0133 PMC8713255

[48] Bhurosy T Niu Z Heckman CJ. Breastfeeding is possible: a systematic review on the feasibility and challenges of breastfeeding among breast cancer survivors of reproductive age. Ann Surg Oncol. 2021;28:3723–35. 32915334 10.1245/s10434-020-09094-1 PMC7947020

[49] Scoccianti C Key TJ Anderson AS. European code against cancer 4th edition: breastfeeding and cancer. Cancer Epidemiol. 2015;39(Suppl 1):S101–6. 26116994 10.1016/j.canep.2014.12.007

[50] Jin E Kang H Son M. Association between breastfeeding and breast, thyroid, and cervical cancer among Korean adult women based on the Korean Genome and epidemiology study: a cohort study. Korean J Women Health Nurs. 2021;27:368–78. 36311452 10.4069/kjwhn.2021.11.29 PMC9328631

[51] Fernández-Aparicio A Schmidt-RioValle J García PA González-Jiménez E. Short breastfeeding duration is associated with premature onset of female breast cancer. Clin Nurs Res. 2022;31:901–8. 35075913 10.1177/10547738211069725

[52] Shield KD Dossus L Fournier A. The impact of historical breastfeeding practices on the incidence of cancer in France in 2015. Cancer Causes Control. 2018;29:325–32. 29464426 10.1007/s10552-018-1015-2

[53] Collaborative Group on Hormonal Factors in Breast Cancer. Breast cancer and breastfeeding: collaborative reanalysis of individual data from 47 epidemiological studies in 30 countries, including 50302 women with breast cancer and 96973 women without the disease. Lancet. 2002;360:187–95. 12133652 10.1016/S0140-6736(02)09454-0

[54] Migliavacca Zucchetti B Peccatori FA Codacci-Pisanelli G. Pregnancy and lactation: risk or protective factors for breast cancer? Adv Exp Med Biol. 2020;1252:195–7. 32816282 10.1007/978-3-030-41596-9_27

[55] Chu YR Kung PT Liu LC. Comparison of quality of life between breast cancer patients treated with and without adjunctive traditional Chinese medicine in Taiwan. Integr Cancer Ther. 2023;22:15347354221150907. 36688414 10.1177/15347354221150907 PMC9893072

[56] Yu Q Xu C Song J Jin Y Gao X. Mechanisms of traditional Chinese medicine/natural medicine in HR-positive breast cancer: a comprehensive literature review. J Ethnopharmacol. 2024;319(Pt 3):117322. 37866466 10.1016/j.jep.2023.117322

[57] Wang W Xu L Shen C. Effects of traditional Chinese medicine in treatment of breast cancer patients after mastectomy: a meta-analysis. Cell Biochem Biophys. 2015;71:1299–306. 25398591 10.1007/s12013-014-0348-z

[58] Cheng Q Huwei B Tao J. Traditional Chinese medicine for bone metastasis of breast cancer: a systematic review and meta-analysis. Eur J Gynaecol Oncol. 2022;43:13–20.

[59] Xia D Li W Tang C Jiang J. Astragaloside IV, as a potential anticancer agent. Front Pharmacol. 2023;14:1065505. 36874003 10.3389/fphar.2023.1065505 PMC9981805

[60] Hung YL Leung SS Chiu SP. Perceptions about traditional Chinese medicine use among Chinese breast cancer survivors: a qualitative study. Cancer Med. 2023;12:1997–2007. 36073533 10.1002/cam4.5046 PMC9883569

[61] Qi F Zhao L Zhou A. The advantages of using traditional Chinese medicine as an adjunctive therapy in the whole course of cancer treatment instead of only terminal stage of cancer. Biosci Trends. 2015;9:16–34. 25787906 10.5582/bst.2015.01019

[62] Tola YO Chow KM Liang W. Effects of non-pharmacological interventions on preoperative anxiety and postoperative pain in patients undergoing breast cancer surgery: a systematic review. J Clin Nurs. 2021;30:3369–84. 33942405 10.1111/jocn.15827

[63] Behzadmehr R Dastyar N Moghadam MP Abavisani M Moradi M. Effect of complementary and alternative medicine interventions on cancer related pain among breast cancer patients: a systematic review. Complement Ther Med. 2020;49:102318. 32147038 10.1016/j.ctim.2020.102318

[64] Zorba P Ozdemir L. The preliminary effects of massage and inhalation aromatherapy on chemotherapy-induced acute nausea and vomiting: a quasi-randomized controlled pilot trial. Cancer Nurs. 2018;41:359–66. 28426542 10.1097/NCC.0000000000000496

[65] Abushukur Y Cascardo C Ibrahim Y Teklehaimanot F Knackstedt R. Improving breast surgery outcomes through alternative therapy: a systematic review. Cureus. 2022;14:e23443. 35481320 10.7759/cureus.23443 PMC9034658

